# Coeliac disease and postpartum depression: are they linked? A two-sample Mendelian randomization study

**DOI:** 10.3389/fpsyt.2024.1312117

**Published:** 2024-07-19

**Authors:** Xiaomeng Yu, Mosong Cheng, Jindan Zheng

**Affiliations:** ^1^ Departments of Obstetrics, Women and Children’s Hospital of Jinzhou, Jinzhou, Liaoning, China; ^2^ Departments of Surgery, Jinzhou Second Hospital, Jinzhou, Liaoning, China; ^3^ Center for Reproductive Medicine, The First Affiliated Hospital of Jinzhou Medical University, Jinzhou, Liaoning, China

**Keywords:** Mendelian randomization, coeliac disease, postpartum depression, depression, SNPs

## Abstract

**Background:**

To explore the potential causal associations between coeliac disease(CD) and postpartum depression(PPD) by using two-sample Mendelian randomization(MR) analysis.

**Methods:**

The IEU OPEN GWAS project was utilized to identify genetic loci strongly associated with CD as instrumental variables (IVs), and MR analysis was performed using inverse variance weighting(IVW), weighted median, weighted model, and MR-Egger. MR analyses were used to examine whether there was a link between CD and PPD, with an OR and 95% CI. Meanwhile, the relationship between CD and depression(DP) was analyzed using MR. The sensitivity analysis was conducted using MR-Egger intercept analysis, Cochran’s Q test, and leave-one-out analysis.

**Results:**

From the GWAS online database, 13 single-nucleotide polymorphisms (SNPs) were chosen as IVs. The IVW results showed a relationship between PPD and a genetically predicted risk of developing CD (OR = 1.022, 95% CI: 1.001–1.044, P = 0.043). However, the presence of DP was not linked with CD (OR=0.991, 95% CI: 0.978–1.003, P=0.151). Potential horizontal pleiotropy was not discovered using MR-Egger intercept analysis (PPD: P=0.725; DP: P=0.785), and Cochran’s Q test for heterogeneity revealed no significant heterogeneity (PPD: P=0.486; DP: P=0.909). A leave-one-out analysis found that individual SNPs had minimal effect on overall causal estimations.

**Conclusion:**

MR research discovered a link between CD and PPD.

## 1 Introduction

Postpartum depression (PPD) is a frequent puerperal mental illness in which women have major depressive symptoms or characteristic depressive episodes throughout the puerperium (1). It is a frequent puerperal mental condition characterized by a persistent and profoundly depressed mood throughout the puerperium, as well as a variety of symptoms such as despair, sorrow, irritability, and even suicidal ideation. These symptoms significantly impair the mother’s capacity to care for her unborn child (2). The aetiology and pathophysiology of PPD remain unknown, and the illness has a worldwide prevalence rate ranging from 7% to 9% (3). It is thought to be closely linked to genetic, neurobiochemical, and psychosocial factors, in addition to maternal helplessness, bad mood, lower energy, and other negative mental characteristics. A recent large-scale clinical study involving over 1 million women from 138 countries discovered that PPD symptoms were most commonly self-reported by women between the ages of 18 and 24, and that women were twice as likely as men to experience depression during childbirth. PPD prevalence decreases with age, reaching a low of 6.5% among persons aged 35 to 39. First-time mothers are more likely to develop comorbid depression than women who have previously given birth. Twin births had a greater symptom load than single births, with 11.3 percent of twin mothers experiencing depressive symptoms compared to 8.3 percent of single-birth mothers. This disparity is especially pronounced in women over the age of 40 (4). A separate study found that PPD can last up to three years, which is far longer than previously thought (5). PPD has a long duration and a significant impact, hurting relationships, families, society, and maternal health and placing mothers and newborns at risk. Identifying people at high risk of having PPD, changing behaviors, and developing preventative measures to minimize and eliminate risk factors for PPD are all effective approaches to reducing the condition’s prevalence.

Coeliac disease (CD), also known as gluten-sensitive enteropathy, is a common immune-mediated inflammatory illness of the small intestine characterized by the body’s sensitivity to dietary gluten and associated proteins in genetically susceptible individuals (6). According to epidemiological research, CD affects around 1% of the global population (7). CD is widespread in most countries at a rate of 1:300–1:70, according to an epidemiological study based on serological testing and biopsy confirmation (8). Another meta-analysis discovered an even higher worldwide frequency of 1.4% based on serological tests and 0.7% based on biopsies (9). Every year, the frequency of CD rises, and it has been associated with a variety of conditions, including unexplained infertility in women, intrauterine growth restriction, and recurrent miscarriage (10). Recent research has found that PPD is frequent in CD women on gluten-free diet GFD, particularly in those with previous menstrual disorders. we suggest screening for PPD in CD for early detection and treatment of this condition (11). Less study has been conducted on the relationship between CD and PPD, and it is still unclear whether one exists.

Mendelian randomization (MR) is a data analysis technique for evaluating etiological inferences in epidemiological studies that is based on whole genome sequencing data and uses genetic variants with strong correlations to exposures as instrumental variables to assess causal associations between exposures and outcomes (12). It is effective at reducing prejudice. In the current study, MR was used to look into any possible causal links between CD and PPD.

## 2 Methods

### 2.1 Study design

We evaluated the probable causal relationship between CD phenotypes and the occurrence of PPD in this study by using the genetic pooled dataset from the genome-wide association study (GWAS) for a two-sample MR analysis with CD-related phenotypes as the exposure factor and the presence of PPD as the outcome. Meanwhile, in the risk factor analysis of PPD, we used depression (DP) as the outcome to investigate whether CD mediates the causal relationship with PPD via DP.

### 2.2 GWAS data source

The exposure variable is celiac disease, and the outcome variables are postpartum depression and depression. All GWAS data are sourced from the IEU OPEN GWAS project (https://gwas.mrcieu.ac.uk/). Celiac disease (ID: finn-b-K11_COELIAC) includes 16,380,438 SNPs, encompassing 1,973 cases and 210,964 controls. Postpartum depression (ID: finn-b-O15_POSTPART_DEPR) includes 16,376,275 SNPs, with 7,604 cases and 59,601 controls. Depression (ID: finn-b-F5_DEPRESSIO) includes 16,380,457 SNPs, with 23,424 cases and 192,220 controls. To ensure ethnic homogeneity, all participants in the sample are of European ancestry, as detailed in **Table 1**.

**Table 1:** Summary of genome-wide association study data in this Mendelian randomization study.

	GWAS ID	Year	Trait	Consortium	Cases/controls	Population
Exposur	finn-b-K11_COELIAC	2021	Coeliac disease	NA	1793/210964	European
Outcomes	finn-b-O15_POSTPART_DEPR	2021	Postpartum depression	NA	7604/59601	European
	finn-b-F5_DEPRESSIO	2021	Depression	NA	23424/192220	European

NA, Not available.

### 2.3 Selection of IVs

**Figure 1:**
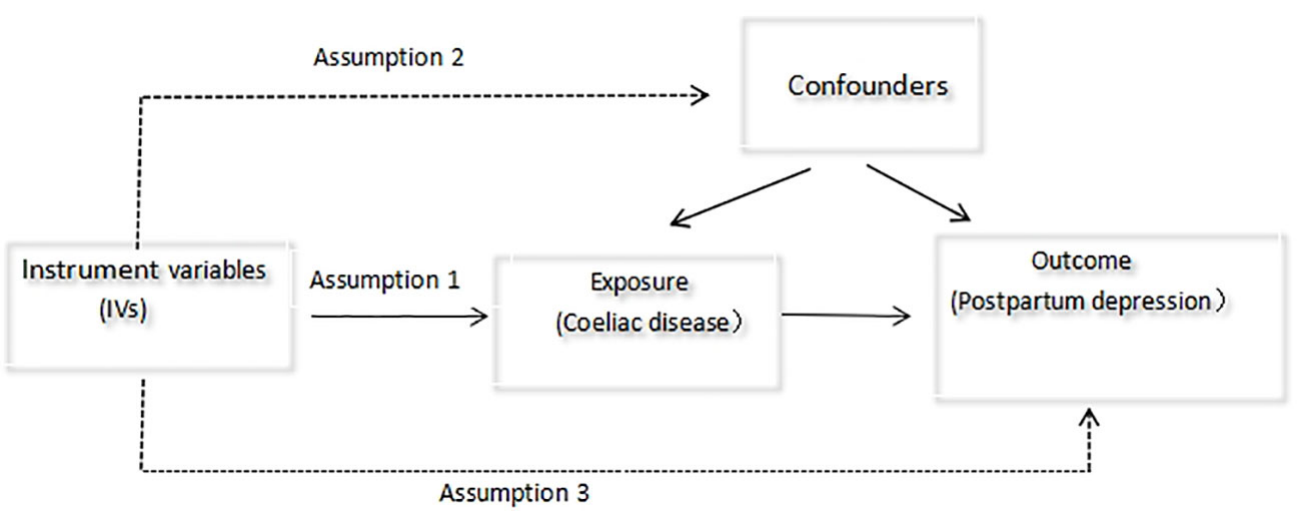
Hypotheses for Mendelian randomization design.

The instrument variables (IVs) in this study were needed to meet the following standards (13): (i) There was no linkage disequilibrium (LD) between single-nucleotide polymorphisms (SNPs), with r2<0.001; (ii) There was no genome-wide significant connection between SNPs and the occurrence of PPD; and (iii) SNPs did not achieve genome-wide significant association with PPD-associated symptoms. The development of puerperal sorrow was not related to SNPs across the genome (P<5×10^-8^) (**Figure 1**). Finally, 13 SNPs associated with CD were included. The F-value was determined using the following method to determine the IVs’ strength in order to rule out any potential weak instrumental variable bias between IVs and exposure factors (14). F = (R^2^/1-R^2^)(n-k-1/K), R^2^= 2X(1-MAF)X(MAF)X2, where N is the exposed GWAS sample size, K is the number of single nucleotide polymorphisms, R2 is the percentage of variation explained by single nucleotide polymorphisms in the exposed database, MAF is the effect allele frequency, and is the allele effect value. When F > 10, weak instrumental variance bias is expected to be less frequent.

### 2.4 Statistical analyses

In our two-sample MR, we used CD as the exposure and PPD and DP as the outcomes. For all studies, the “TwoSampleMR” analytic technique was utilized. For all analyses in R (4.3.1), the “TwoSampleMR” package, version 0.5.7, was utilized. The outcome-related SNPs were removed using PhenoScanner V2. The outliers were removed using MR-PRESSO. To study the causal influence, inverse variance weighting (IVW), weighted median, weighted model, and MR-Egger techniques were utilized. Odds ratios (OR) and 95% confidence intervals (CI) were calculated to assess the probable causal relationship between CD and the chance of developing prenatal depression. Possibility of a causal relationship. The sensitivity analysis was conducted using MR-Egger intercept analysis, Cochran’s Q test, and leave-one-out analysis.

## 3 Results

### 3.1 IVs

In this work, the PhenoScanner database was utilized to search for phenotypes in line with the screening criteria for instrumental factors, and a total of 13 SNPs that were closely connected to one another without chain disequilibrium were originally screened out from the CD exposure data set. There were no SNPs with PPD significance, and the F-value statistic for 13 SNPs was larger than 10, suggesting that there were no weak instrumental factors in the MR analysis, and the MR-PRESSO analysis did not disclose any outliers. Finally, 13 SNPs were used as IVs (P<5×10^-8^, r2<0.001, clumping distance = 10,000 kb) to assess the relationship between CD and PPD.

### 3.2 Causal association between CD and PPD

**Figure 2:**
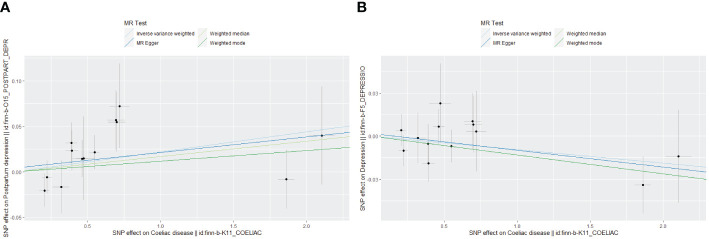
Scatter plot of Mendelian randomization effect size for causal associations. **(A)** Coeliac disease and postpartum depression; **(B)** Coeliac disease and depression.

**Figure 3:**
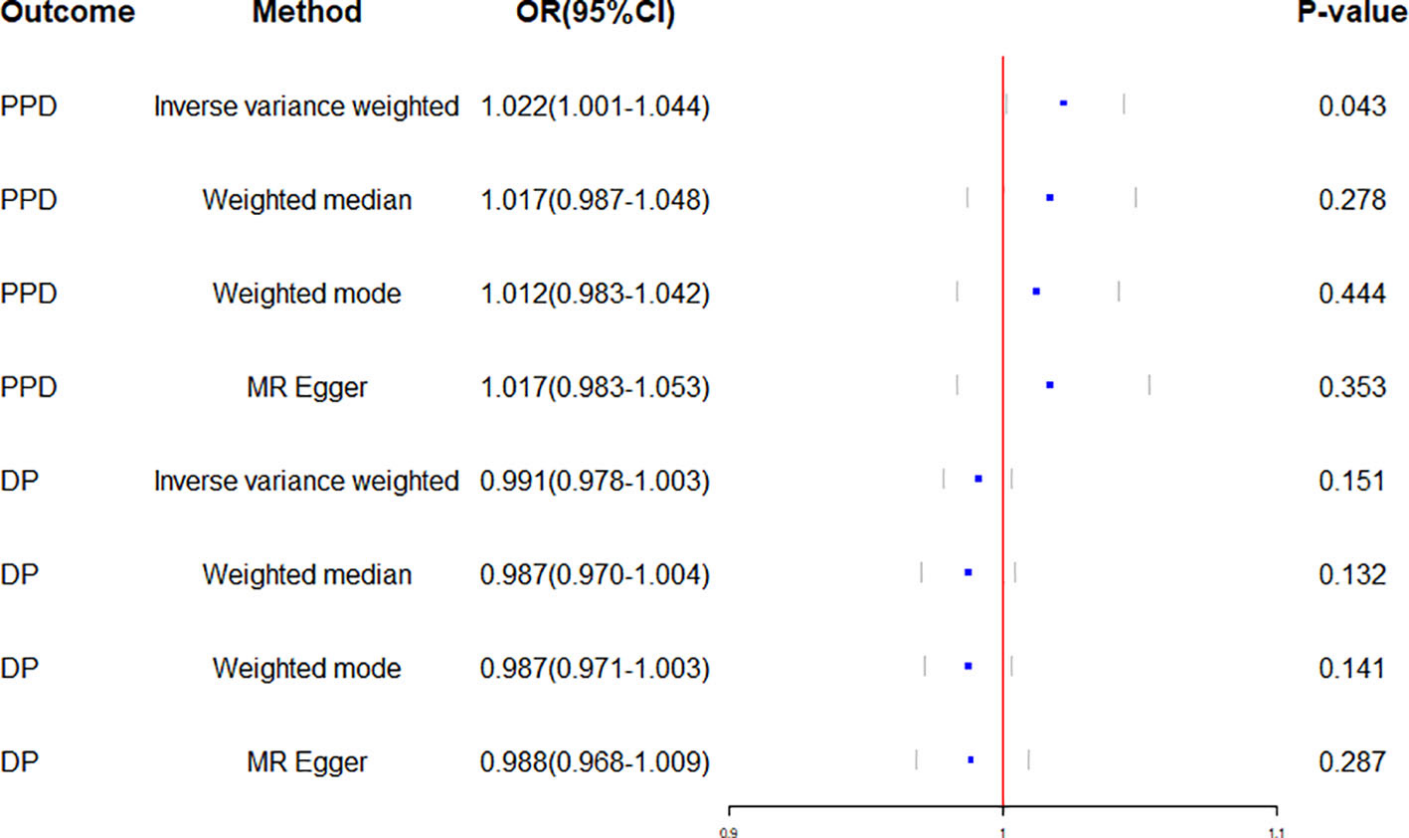
Forest plot of Mendelian randomization effect sizes for causal associations. PPD, Postpartum depression; DP, Depression; OR, Odds ratio; CI, Confidence interval.

**Figure 4:**
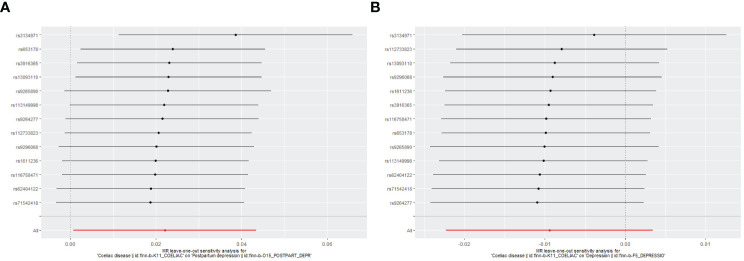
Leave-one-out plot to assess if a single variant is driving the association. **(A)** Coeliac disease and postpartum depression; **(B)** Coeliac disease and depression.

Genetically predicted CD and PPD were shown to have a positive and statistically significant causative relationship using the IVW technique in MR analysis (OR = 1.022, 95% CI: 1.001–1.044, P = 0.043). Three additional analysis techniques Although the three methods did not support a causal relationship between CD and PPD, the four methods of IVW, weighted median, weighted mode, and MR-Egger were consistent in the direction of the results. These methods were also used to confirm the robustness of the results. The four studies’ findings all pointed in the same direction (OR > 1). Additionally, scatter plots demonstrated the general consistency of the regression lines discovered for the genetic prediction of CD on the risk of PPD (**Figure 2A**). The lack of possible horizontal pleiotropy detected by MR-Egger intercept analysis (P = 0.725) suggests that IVs do not significantly alter outcomes through mechanisms other than exposure. No significant heterogeneity was found in the Cochran’s Q test for heterogeneity (P = 0.486) (**Table 2**; **Figure 3**). No specific single nucleotide polymorphism had an impact on the overall causal estimate, according to a leave-one-out analysis (**Figure 4A**). The preceding sensitivity analysis demonstrates that the effect OR values derived from the IVW method are quite robust.

**Table 2:** The effects of coeliac disease on the risk of postpartum depression and depression as determined by Mendelian randomization.

Exposure	Outcomes	Methods	IVs(n SNPs)	Beta	SE	*P*-value	OR	95% CI	Pleiotropy *P*-value	Heterogeneity *P*-value
Coeliac disease	Postpartum depression	IVW	13	0.022	0.0110.016	0.043	1.022	1.001,1.044	0.725	0.486
		Weighted median	13	0.017	0.015	0.278	1.017	0.987,1.048		
		Weighted mode	13	0.012	0.018	0.444	1.012	0.983,1.042		
		MR-Egger	13	0.017		0.353	1.017	0.983,1.053		
	Depression	IVW	13	-0.009	0.007	0.151	0.991	0.978,1.003	0.785	0.909
		Weighted median	13	-0.013	0.009	0.132	0.987	0.970,1.004		
		Weighted mode	13	-0.013	0.008	0.141	0.987	0.971,1.003		
		MR-Egger	13	-0.012	0.010	0.287	0.988	0.968,1.009		

SNP, single nucleotide polymorphisms; IVs, instrumental variables; OR, odds ratio; CI, confidence interval; SE, standard error, n, number.

### 3.3 Association between CD and DP

Genetically predicted CD and DP may not be causally related, as the IVW method showed in MR investigations (OR=0.991, 95% CI: 0.978–1.003, P=0.151). The other three methods confirmed that there is no causal relationship between CD and DP. Similar scatter plots illustrated how the regression lines produced for the genetically predicted risk of CD and DP were generally consistent (**Figure 2B**). The MR-Egger intercept analysis failed to detect any potential horizontal pleiotropy (P = 0.785). No significant heterogeneity was found using the Cochran’s Q test (P = 0.909). The leave-one-out analysis failed to identify any particular SNPs that had an effect on the overall causal estimates (**Figure 4B**).

## 4 Discussion

Identifying the etiology of PPD is important for its prevention, diagnosis, and treatment. This study looked into the connection between CD and PPD using MR analysis. The results of the MR investigation showed a clear causal link between CD and PPD.

The association between CD and PPD has not been made clear till now. According to certain studies, people with CD can experience neurological or psychological symptoms such as anxiety, sadness, headache, peripheral neuropathy, ataxia, and epilepsy (15, 16). These studies had some drawbacks, including small sample numbers, retrospective data, the possibility of an inaccurate CD diagnosis, and referral bias from tertiary care facilities. Some of these investigations used the presence of anti-antimelanocortin antibodies to make the diagnosis rather than duodenal histological signs or more specific autoantibodies. Concerning the connection between CD, DP, and epilepsy, there is conflicting information. Due to the superior research design, we were confident in proving causality in addition to bias in the current study employing MR techniques. It was difficult for prior observational studies to prevent bias due to confounding risk factors. This was the first study to do an MR analysis between CD and PPD risk. According to the research, CD may have a significant causal link that increases the risk of PPD.

Out of the many recognized potential risk factors for PPD, a past history of depression in the perinatal or non-perinatal era has the greatest impact and the strongest correlation with PPD (17, 18). Depression that was present at high levels during the pregnancy may persist during labor and delivery (19). More than half of women with histories of prenatal depression (AD) also had postpartum depression, according to a study (20). On the other hand, a review of the literature showed that postpartum depression affected more than a third (39%) of women with AD (21). In order to exclude the influence of depression as a risk factor on the result, the correlation between CD and depression was examined in this study utilizing MR analysis. The results of the MR investigation showed that CD and DP did not clearly share a causal relationship. It was also proven that CD and PPD are causally connected.

This study’s strength stems from the fact that it is the first to use data from a substantial population sample in a systematic genetic technique to look into the potential of a connection between CD and the prevalence of PPD. The stability of the data was also confirmed using a number of statistical approaches, such as inverse variance weighting, weighted median, maximum likelihood ratio, MR-Egger regression analysis, and the MR-PRESSO method. However, this study also has limitations, such as the inclusion of populations from Europe, which may reduce population stratification bias, but the reliability of extrapolation to other ethnic groups may be insufficient. Therefore, it is necessary to further study the relationship between CD and PPD in other ethnic groups.

Ultimately, the present study employed two-sample MR research to carefully assess the potential causal associations between CD and the incidence of PPD at the genetic level. The results indicated that since CD was positively correlated with the chance of developing PPD, prevention of CD could have a prophylactic impact on the development of PPD.

## Data availability statement

The raw data supporting the conclusions of this article will be made available by the authors, without undue reservation.

## Author contributions

XY: Writing – original draft. MC: Writing – original draft, Data curation, Software. JZ: Funding acquisition, Visualization, Writing – original draft, Writing – review & editing.
